# SYK is activated by mutated MYD88 and drives pro-survival signaling in MYD88 driven B-cell lymphomas

**DOI:** 10.1038/s41408-020-0277-6

**Published:** 2020-01-31

**Authors:** Manit Munshi, Xia Liu, Jiaji G. Chen, Lian Xu, Nickolas Tsakmaklis, Maria G. Demos, Amanda Kofides, Maria Luisa Guerrera, Cristina Jimenez, Gloria G. Chan, Zachary R. Hunter, M. Lia Palomba, Kimon V. Argyropoulos, Kirsten Meid, Andrew Keezer, Joshua Gustine, Toni Dubeau, Jorge J. Castillo, Christopher J. Patterson, Jinhua Wang, Sara J. Buhrlage, Nathanael S. Gray, Steven P. Treon, Guang Yang

**Affiliations:** 1 Bing Center for Waldenstrom’s Macroglobulinemia, Dana Farber Cancer Institute and Harvard Medical School, Boston, MA USA; 2 Department of Medical Oncology, Dana Farber Cancer Institute and Harvard Medical School, Boston, MA USA; 3 Lymphoma Service, Memorial Sloan Kettering Cancer Center, New York, NY USA; 4 Department of Biological Chemistry and Molecular Pharmacology, Harvard Medical School, Boston, MA USA

**Keywords:** Cell signalling, Targeted therapies

## Abstract

Activating MYD88 mutations promote pro-survival signaling through BTK and HCK, both targets of ibrutinib. Despite high response rates, complete responses to ibrutinib are lacking, and other MYD88 triggered pro-survival pathways may contribute to primary drug resistance. B-cell receptor (BCR) signaling has been observed in lymphomas driven by mutated MYD88, even without activating the BCR pathway mutations. We identified activated SYK (p-SYK), a component of BCR in complex with MYD88 in *MYD88*-mutated WM and ABC DLBCL lymphoma cells. Confocal microscopy confirmed co-localization of MYD88 with SYK in *MYD88*-mutated cells. Knockdown of MYD88 or use of a MYD88 signaling inhibitor abrogated SYK activation, while expression of mutated but not wild-type *MYD88* amplified p-SYK in *MYD88*-mutated and wild-type lymphoma cells. Knockdown of SYK or use of inhibitors targeting SYK blocked p-STAT3 and p-AKT signaling in *MYD88*-mutated cells. Cell viability analysis showed that combining ibrutinib and SYK inhibitors triggered synthetic killing of *MYD88*-mutated lymphoma cells. Our findings extend the spectrum of mutated *MYD88* pro-survival signaling to include SYK directed BCR cross talk in *MYD88*-mutated lymphomas. Targeting SYK in combination with ibrutinib produces synthetic lethality, providing a framework for the clinical investigation of ibrutinib with SYK inhibitors in *MYD88*-mutated lymphomas.

## Introduction

Activating mutations in *MYD88* promote Myddosome self-assembly and trigger Toll-like receptor (TLR) and IL-1 receptor (IL-1R) signaling^1–5^. Downstream components of mutated *MYD88* signaling include BTK and IRAK4/IRAK1 that trigger NFκB pro-survival signaling, as well as HCK transactivation that activates BTK itself, AKT and ERK^1–6^. Both BTK and HCK are targets of ibrutinib, a pleiotropic kinase inhibitor that is active in patients with *MYD88*-mutated B-cell malignancies, including Waldenstrom’s macroglobulinemia (WM), ABC subtype of diffuse large B-cell lymphoma (DBLCL), and primary CNS lymphoma (PCNSL)^7–10^. Despite high rates of response to ibrutinib, complete responses are lacking, and other mutated MYD88 triggered pathways may contribute to sustained pro-survival signaling and primary drug resistance.

Activation of B-cell receptor (BCR) signaling is common among *MYD88*-mutated B-cell malignancies. Activating *CD79A/B* mutations are frequently present in ABC DLBCL and PCNSL and can trigger SYK-mediated downstream signaling^2,11,12^. In contrast to these aggressive entities, *CD79A/B* activating mutations are uncommon in WM with a reported incidence of 3–12%, and have been associated with transformed WM disease^13–16^. Despite the absence of activating BCR mutations, active B-cell receptor (BCR) signaling has been observed in WM and ABC DLBCL patients, and could contribute to pro-survival signaling^17–19^. We therefore have sought to clarify whether mutated *MYD88* could account for chronic BCR signaling in *MYD88-*mutated lymphoma cells. While BTK, a component of the BCR signalosome is triggered by mutated *MYD88*, our previous work showed that the upstream BCR signaling member SYK was activated in *MYD88*-mutated WM patient cells^17^. Moreover, the SYK inhibitor fostamatinib triggered apoptosis of *MYD88*-mutated WM cells, suggesting an important function for SYK in MYD88 pro-survival signaling^20^. We therefore investigated a role for SYK as a mediator of BCR activation in *MYD88*-mutated lymphomas. Knowledge of MYD88-directed SYK signaling could prompt development of novel therapeutic approaches aimed at extinguishing other routes for MYD88-directed pro-survival signaling beyond ibrutinib and other BTK inhibitors.

## Methods

### Cell lines and reagents

*MYD88 L265P* (*MYD88*^*L265P*^) expressing WM (BCWM.1 and MWCL-1), ABC DLBCL (TMD8, HBL-1, OCI-Ly3) and wild-type *MYD88* (*MYD88*^*WT*^) GCB DLBCL (OCI-Ly7, OCI-Ly19), Burkitt’s lymphoma (Ramos), as well as multiple myeloma (RPMI-8226) cells were used in the studies. The identities of the cell lines used in this study were confirmed via STR profiling with GenePrint® 10 System (Promega, Madison, WI) at The Molecular Diagnostic Laboratory at Dana-Farber Cancer Institute. All cell lines are routinely tested to exclude mycoplasma contamination. TMD8 (Y196H) and HBL-1 (Y196F) carry *CD79B* activating mutations, while OCI-LY3 is homozygous for *MYD88*^*L265P*^. All cell lines were cultured as previously described^5,6^. The BTK inhibitor ibrutinib, and SYK inhibitors tamatinib (R406), the active metabolite of fostamatinib^21^, and entospletinib (GS-9973)^22^ were obtained from Selleck Chemicals (Houston, TX). MYD88 inhibitor and control peptides were obtained from Novus Biologicals (Littleton, CO), and used as before in signaling experiments^1,5^.

### Patient samples and treatments

Mononuclear cells from freshly obtained WM patient’s bone marrow (BM) aspirates were isolated using Ficoll-Paque™ PLUS Media (GE Healthcare™) and treated with either ibrutinib, SYK inhibitor tamatinib or entospletinib, or combination of ibrutinib and a SYK inhibitor. Apoptosis analyses were performed on *MYD88* genotyped CD19-gated lymphoplasmacytic cells (LPCs) following overnight treatment of BM mononuclear cells, as previously described^5,6,23^. Subject participation was approved by the Harvard Cancer Center/Dana-Farber Cancer Institute Institutional Review Board, and all participants provided written consent for sample use.

### Lentiviral knockdown and expression studies

Knockdown of endogenous MYD88 in *MYD88*^*L265P*^ expressing BCWM.1 WM and *MYD88*^*L265P*^ and *CD79B*^*Y196H*^ expressing TMD8 cells was undertaken, as well as expression of MYD88^WT^ or MYD88^L265P^ proteins in BCWM.1, OCI-Ly7 or Ramos cells following lentiviral transduction as previously described^5,6^. Knockdown of SYK was performed using lentiviral vector system as previously described that targeted the following sequences: shRNA-1 (5′-GGGAAGAATCTGAGCAAAT-3′); shRNA-2 (5′-GGATCAAAGACAAATGGAA-3′). Following lentiviral transduction on day 5, SYK knockdown cells were selected by flow sorting of GFP-positive cells and analyzed in cell viability and signaling studies.

### Signaling studies

PhosFlow and immunoblotting studies were performed as previously described^5,6^ using antibodies to p-SYK(Y525/Y526) (R&D Systems, MN); p-BTK(Y223), p-IRAK1(T209) (Abcam, MA); SYK, p-STAT3(Y705), STAT3, p-AKT(S473), AKT, BTK, IRAK1, p-IRAK4(T345/S346), IRAK4, and Alexa Fluor^®^ 647-conjugated p-SYK(Y525/Y526) (Cell Signaling Technologies, MA). Alexa Fluor^®^ 488-conjugated α-tubulin antibody (Cell Signaling Technologies) was used as an internal control for p-SYK levels among different cell lines. GAPDH antibody (Santa Cruz Biotechnology, TX) was used as a loading control for immunoblotting. Cell lines or WM patient BM mononuclear cells were treated with inhibitors for 1–2 h before signaling studies.

### Co-immunoprecipitation experiments

Co-immunoprecipitation studies were performed as previously described^5^ using anti-MYD88 antibody (Santa Cruz Biotechnology) and SYK, p-SYK(Y525/Y526) antibodies (Cell Signaling Technologies). Briefly, cells were lysed with Co-IP buffer (Thermo Fisher Scientific) supplemented with 1 mM sodium orthovanadate, 10 mM NaF, 1 × protease inhibitors cocktail for 15 min on ice, and then centrifuged at 2600 × *g* for 5 min. Supernatants (2 mg total protein) were incubated with 2–4 µg of antibodies at 4 °C for 30 min, followed by incubation with protein A/G-coated magnetic beads (EMD Millipore) for another 30 min at 4 °C. After samples were washed four times with ice-cold lysis buffer on a magnetic stand, proteins were eluted using SDS-PAGE loading buffer for further analysis.

### Immunofluorescence staining and confocal microscopy

BCWM.1, MWCL-1, and TMD8 cells were spun onto glass slides with Cytospin™ 4 Cytocentrifuge (Thermo Fisher Scientific) at 800 rpm for 5 min. Cells were fixed with 4% paraformaldehyde in PBS for 20 min, washed twice with PBS, permeabilized with 0.25% Triton X-100 in PBS for 15 min, and blocked with blocking buffer (5% BSA and 0.1% Triton X-100 in PBS) for 1 h. Cells were then incubated with anti-MYD88-Alexa Fluor^®^ 488 and anti-SYK-Alexa Fluor^®^ 647 antibodies (Santa Cruz Biotechnology) overnight at 4 °C. The slides were washed five times with 1× PBS and counterstained with mounting media containing DAPI (4′,6-diamidino-2-phenylindole dihydrochloride) (Thermo Fisher Scientific) and imaged using Leica SPE Confocal Microscope (Leica Microsystems) using an ACS APO 63×/1.30 oil immersion lens. Images were analyzed using Leica Application Suite X software.

### Cytotoxicity studies

The CellTiter-Glo® Luminescent cell viability assay (Promega, Madison, WI) was used to assess the dose response of inhibitors alone or in combination^5,6^. Cells were seeded into 384 or 96-well plates with the EL406 Combination Washer Dispenser (BioTek Instruments, Inc.), and inhibitors were injected into culture media with the JANUS Automated Workstation (PerkinElmer Inc., Waltham, MA, USA). Cells were incubated with inhibitors for 72 h at 37 °C. Luminescent measurements to assess cell viability were performed using the 2104 Envision® Multilabel Reader (PerkinElmer Inc.). Drug interactions were assessed by CalcuSyn 2.0 software (Biosoft, Cambridge, UK) based on Chou TC^24^.

### Reproducibility and statistical analysis

Sample size of experiments and the number of experiments performed are depicted in the figure legends. The statistical significance of differences was analyzed using one-way ANOVA with Tukey’s multiple comparisons test by Prism software. Differences were considered significant when *p* < 0.05. Error bars denote standard deviation.

## Results

### SYK is activated in *MYD88*-mutated lymphoma cells, and its activation is regulated by MYD88

**Fig. 1:**
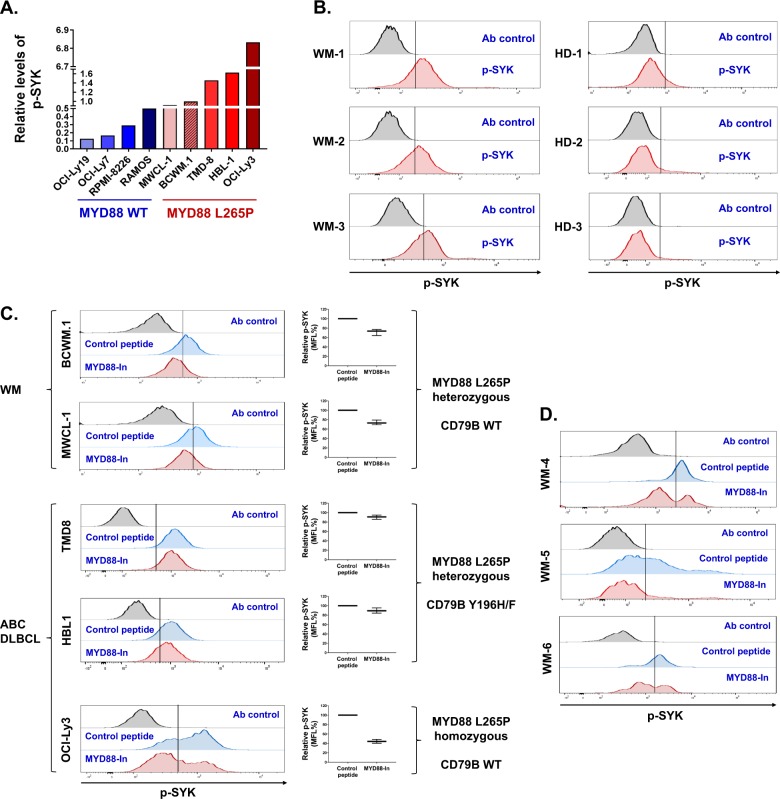
SYK is activated in *MYD88*-mutated lymphoma cells, and its activation is regulated by MYD88. Relative levels of p-SYK (pY525-pY526) in *MYD88*-mutated and wild-type cell lines. Studies were performed twice, and representative experimental set is shown (**a**). Relative levels of p-SYK (pY525-pY526) in three *MYD88*-mutated primary WM cells and three healthy donor B cells (**b**). The results from PhosFlow studies for p-SYK (pY525-pY526) following treatment with a MYD88 inhibitor or control peptide in lymphoma cells carrying only mutated *MYD88*, or mutated *MYD88* and *CD79B*. Representative histograms and relative median fluorescence intensity from three independent experiments are shown (**c**). Findings from PhosFlow studies for p-SYK (pY525-pY526) following treatment of primary *MYD88*-mutated WM cells from two patients with a MYD88 inhibitor or control peptide (**d**).

A cross cell line comparison of the phosphorylation levels of SYK (pY525-pY526) showed higher levels of activated SYK in MYD88-mutated versus wild-type cell lines, while ABC DLBCL cells (TMD8, HBL-1) with both *MYD88* and *CD79B* mutations expressed higher levels of phosphorylated SYK versus *MYD88* only mutated WM cells (Fig. 1a). The highest p-SYK levels were observed in OCI-Ly3 ABC DLBCL cells that are homozygous for *MYD88*^*L265P*^ (Fig. 1a). More pronounced SYK phosphorylation was also identified in primary *MYD88*-mutated WM cells when compared with healthy donor peripheral B cells (Fig. 1b). We next performed PhosFlow studies for SYK in *MYD88*-mutated lymphoma cells following treatment with a MYD88 inhibitor or control peptide. Akin to our previous findings for NFkB signaling^1,5^, these studies also showed that phosphorylation of SYK at Tyrosine 525 and 526 (pY525-pY526) was reduced following treatment with a MYD88 peptide inhibitor in both *MYD88*-mutated WM and ABC DLBCL cells (Fig. 1c). Noteworthy, SYK phosphorylation was more robustly reduced in the WM cell lines (BCWM.1, MWCL-1), as well as the ABC DLBCL cell line OCI-Ly3 that carry only the *MYD88*^*L265P*^ mutation, but negligible in ABC DLBCL cells (TMD8, HBL-1) with both *MYD88* and *CD79B* activating mutations (Fig. 1c). Treatment of primary *MYD88*-mutated WM cells with the MYD88 peptide inhibitor also blocked SYK phosphorylation (Fig. 1d).

**Fig. 2:**
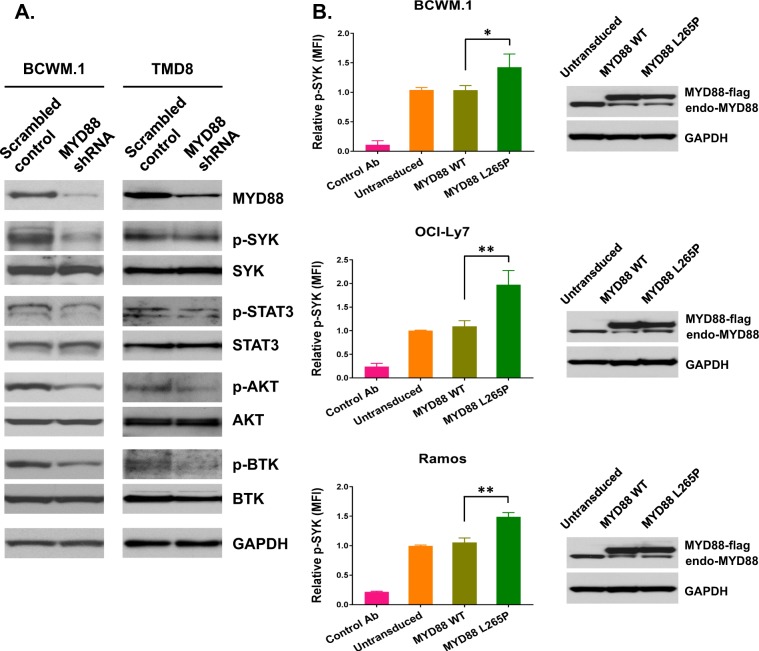
SYK is a downstream target of mutated *MYD88*. Impact of MYD88 knockdown in *MYD88*-mutated BCWM.1 WM and *MYD88/CD79B* mutated TMD8 ABC DLBCL cells on p-SYK (pY525-pY526), p-STAT3 (pY705), p-AKT (pS473), and p-BTK (Y223) by western blot analysis. Studies were performed twice, and representative experimental set is shown (**a**). Findings from transduction experiments showing p-SYK (pY525-pY526) levels following expression of mutated (L265P) or wild-type MYD88 in *MYD88*-mutated BCWM.1 cells, and *MYD88* wild-type OCI-LY7 and Ramos cells by PhosFlow analysis. GADPH was used to depict uniform protein loading. *p < 0.05; **p < 0.001. The protein levels of transduced N-terminal flag tagged MYD88 WT and MYD88 L265P are shown by western blot analysis. Studies were performed in triplicate for each experimental set, and representative experimental set of two independent experiments is shown (**b**).

To further explore the possibility that SYK was activated by mutated *MYD88*, we performed a knockdown of MYD88 in *MYD88-*mutated BCWM.1 WM cells and *MYD88/CD79B* mutated TMD8 ABC DLBCL cells. While these experiments showed a marked reduction in phosphorylation of SYK (pY525-pY526), and the SYK downstream components STAT3 (pY705) and AKT (pS473) in BCWM.1, these changes were not observed in TMD8 cells, consistent with our prior findings using MYD88 inhibitor peptides (Fig. 2a). As shown in our prior studies, p-BTK (Y223) expression was decreased by MYD88 knockdown in both BCWM.1 and TMD8 cells^5^. Importantly, the expression of MYD88^L265P^ but not wild-type MYD88 increased phosphorylated SYK in *MYD88*-mutated BCWM.1, as well as in *MYD88* wild-type OCI-Ly7 and Ramos cell lines (Fig. 2b).

### Activated SYK is a component of the “Myddosome” signaling complex in *MYD88*-mutated lymphoma cells

**Fig. 3:**
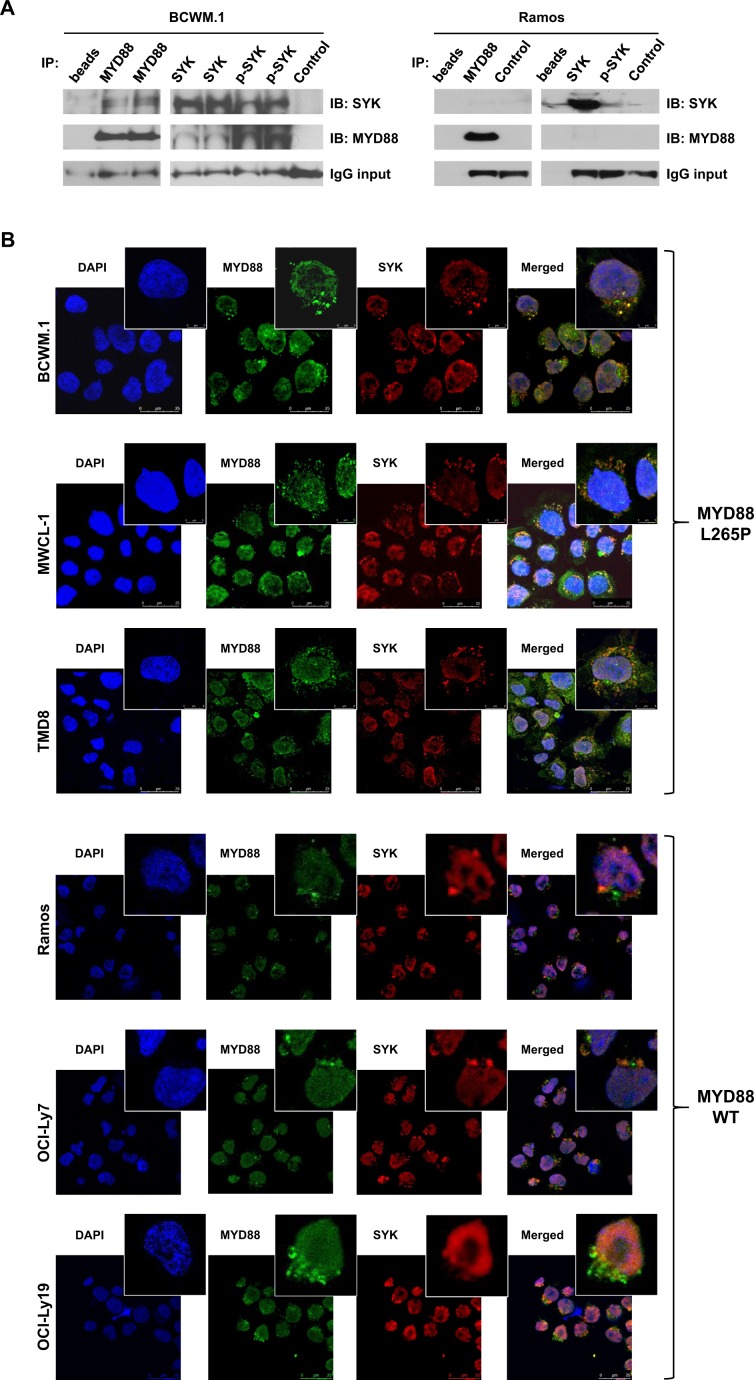
Activated SYK is a component of the “Myddosome” signaling complex in *MYD88*-mutated lymphoma cells. Western blots showing SYK pulldown by MYD88 antibody and MYD88 pulldowns by SYK and p-SYK antibodies in MYD88-mutated and wild-type cells in co-IP experiments. Experiments were run using 2 mg of total protein in duplicated samples, and IgG levels used to demonstrate uniform antibody addition for pull-down experiments (**a**). Confocal microscopy images showing nuclear staining by DAPI (blue); MYD88 staining by anti-MYD88-Alexa Fluor® 488 (green); and SYK staining by anti-SYK-Alexa Fluor® 647 (red) antibodies. MYD88 and SYK protein co-localization appears in the cytoplasm (yellow) with a punctate staining pattern following merging of MYD88 and SYK staining images in MYD88-mutated BCWM.1 and MWCL-1 WM cells, and TMD8 ABC DLBCL cells, but not in MYD88 wild-type Ramos Burkitt’s lymphoma cells, or OCI-Ly7 and OCI-Ly19 GCB DLBCL cells (**b**). Studies were performed twice, and representative experimental set is shown.

Since SYK was activated by mutated *MYD88*, we next sought to clarify if SYK and p-SYK were present in the “Myddosome” signaling complex in *MYD88*-mutated lymphoma cells. We therefore performed co-immunoprecipitation (Co-IP) experiments using a MYD88-binding antibody, as well as reverse Co-IP experiments using both SYK and p-SYK antibodies in *MYD88*-mutated BCWM.1 and *MYD88* wild-type Ramos cells. The MYD88 antibody effectively pulled down SYK in Co-IP experiments, and both SYK and p-SYK antibodies pulled down MYD88, with more robust pulldown of MYD88 by activated SYK in *MYD88-*mutated BCWM.1 cells (Fig. 3a). Conversely, both SYK and p-SYK antibodies failed to pull down MYD88 in *MYD88* wild-type Ramos cells (Fig. 3a). As expected, the IP of p-SYK did not reveal SYK itself in line with the results shown in Fig. 1a that showed lack of p-SYK in MYD88 wild-type cells. Further to these findings, we performed cellular co-localization experiments using immunofluorescent (IF) with antibodies for MYD88 and SYK in both *MYD88*-mutated and wild-type lymphoma cells. By confocal microscopy, IF staining showed that MYD88 and SYK co-localized in the cytoplasm with a punctate staining pattern in *MYD88*-mutated BCWM.1, MWCL-1 and TMD8 cells (Fig. 3b), while the MYD88 showed less punctate staining pattern and not co-localized with SYK in *MYD88* wild-type Ramos, OCI-Ly7, and OCI-Ly19 cells (Fig. 3b).

### Activated SYK promotes STAT3 and AKT signaling as an intermediary of mutated *MYD88*

**Fig. 4:**
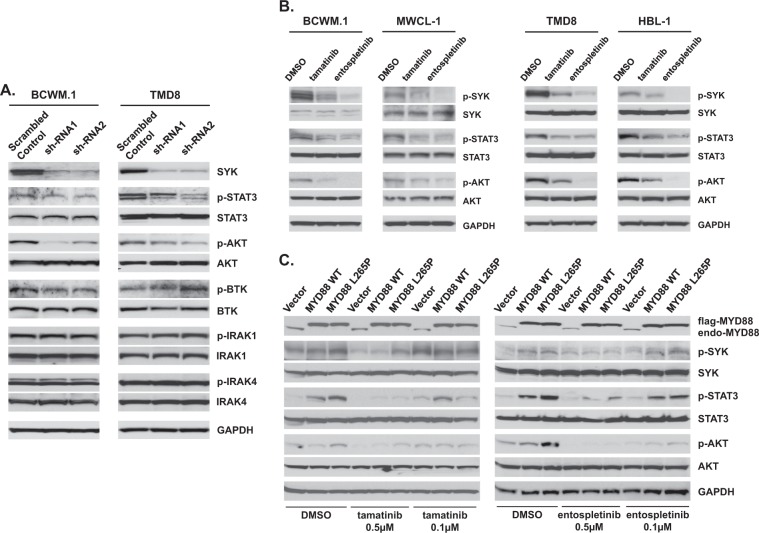
Activated SYK promotes STAT3 and AKT signaling as an intermediary of mutated *MYD88*. Impact of SYK knockdown by lentiviral transduction (**a**) or use of SYK inhibitors tamatinib (TAM) or entospletinib (ENTO) (**b**) on p-STAT3 (Y705), p-ATK (S473), p-BTK (Y223), p-IRAK1(T209), and p-IRAK4(T345/S346) in *MYD88*-mutated BCWM.1 WM and *MYD88/CD79B* mutated TMD8 ABC DLBCL cells. The results from experiments assessing the impact of mutated (L265P) or wild-type MYD88 expression on p-SYK (pY525-pY526), p-STAT3 (pY705), and p-AKT (pS473) in the presence or absence of SYK inhibitors (**c**). GAPDH was used to depict uniform protein loading. Studies were performed twice, and representative experimental set is shown.

To explore the downstream events of activated SYK in *MYD88*-mutated WM cells, we next knocked down SYK using two lentiviral vectors in BCWM.1 cells. Knockdown of SYK reduced the phosphorylation of STAT3 (pY705) and AKT (pS473), while only modest or no changes in BTK, IRAK1 and IRAK4 phosphorylation were observed (Fig. 4a). In addition to these experiments, we treated *MYD88*-mutated WM and ABC DLBCL cells with tamatinib or entospletinib, and examined changes in the phosphorylation levels of SYK, STAT3, and AKT. As expected, tamatinib as well as entospletinib effectively blocked the phosphorylation of SYK, and its downstream components STAT3 and AKT in *MYD88* only mutated WM cells, as well as in ABC DLBCL cell lines carrying both *MYD88* and *CD79B* activating mutations (Fig. 4b). To further clarify the downstream signaling pathways impacted by SYK in *MYD88*-mutated WM cells, we engineered BCWM.1 cells to express vector only, wild-type MYD88 or MYD88 L265P and treated them with the SYK inhibitors tamatinib or entospletinib. The increased phosphorylation of SYK (pY525-pY526), as well as the known downstream signaling components STAT3 (pY705) and AKT (pS473) induced by the expression of MYD88^L265P^ were blocked by both SYK inhibitors in a dose-dependent manner (Fig. 4c).

### SYK supports growth and survival of *MYD88*-mutated lymphoma cells

**Fig. 5:**
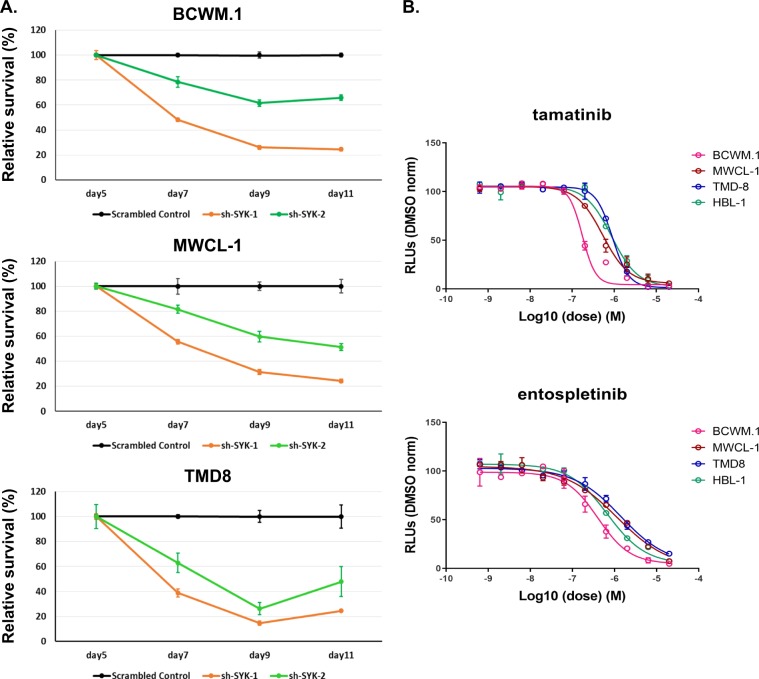
SYK supports growth and survival of *MYD88*-mutated lymphoma cells. Impact of SYK knockdown on the cell growth and survival of *MYD88*-mutated BCWM.1, MWCL-1, and *MYD88/CD79B* mutated TMD8 ABC DLBCL cells was determined by CellTiter-Glo^®^ luminescent cell viability assay following cell sorting for GFP+ cells at day 5 of lentiviral vector transduction. Cell viability was tracked from day 5 to day 11 following lentiviral transduction (**a**). Dose–response curves for the SYK inhibitors tamatinib and entospletinib in *MYD88*-mutated WM (BCWM.1, MWCL-1) and *MYD88/CD79B* mutated ABC DLBCL (TMD8, HBL-1) cells (**b**). Studies were performed in triplicate for each experimental set, and representative experimental set of two independent experiments is shown.

Since SYK activation is directly caused by mutated MYD88 and mediates its downstream STAT3 and AKT signaling, we next sought to clarify the importance of SYK in supporting cell growth and survival in *MYD88*-mutated lymphoma cells. We therefore assessed the cell growth and survival of *MYD88*-mutated WM and ABC DLBCL cells, including *MYD88/CD79B* mutated TMD8 and HBL-1 cells using CellTiter-Glo® Luminescent cell viability assay following SYK knockdown by lentiviral transduction. GFP-positive cells were sorted on day 5 following lentiviral transduction, and the cell growth and survival were determined every 2 days until day 11. Compared with scrambled control, knockdown of SYK reduced cell growth and survival in BCWM.1, MWCL-1, and TMD8 cells (Fig. 5a). Moreover, treatment of *MYD88*-mutated WM and *MYD88/CD79B* ABC DLBCL cell lines with tamatinib or entospletinib showed killing at pharmacologically achievable levels for either agent (Fig. 5b)^21,25^.

### The combined inhibition of BTK and SYK produces synergistic lethality in *MYD88*-mutated lymphoma cells

**Fig. 6:**
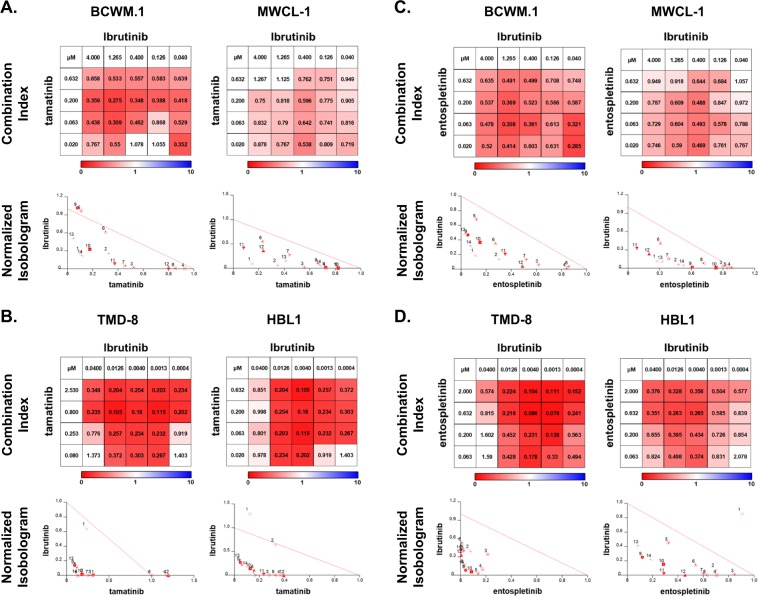
The combined inhibition of BTK and SYK produces synergistic lethality in *MYD88*-mutated lymphoma cells. Evaluation for the combination of ibrutinib with the SYK inhibitors tamatinib (R406) (**a, b**) or entospletinib (GS-9973) (**c, d**) was undertaken in *MYD88*-mutated WM (BCWM.1, MWCL-1) and *MYD88/CD79B* mutated ABC DLBCL (TMD8, HBL-1) cells. The findings from combination index (CI) and normalized isobologram analyses are depicted. CI<1 or the dots under the oblique line in the isobologram plots indicate a synergistic effect for the combination (**a**–**d**). Studies were performed twice, and representative experimental set is shown.

Since SYK provides a divergent stream of pro-survival signaling from previously identified mutated *MYD88* driven BTK and HCK, both targets of ibrutinib, we next sought to examine the combined effects of ibrutinib and either tamatinib or entospletinib. The combination of ibrutinib with either SYK inhibitor produced increased lethality in *MYD88*-mutated BCWM.1 and MWCL-1 WM cells, as well as *MYD88/CD79B* mutated TMD8 and HBL-1 ABC DLBCL cells. Combination index (CI) and normalized isobologram analysis indicated ibrutinib and SYK inhibitor interactions were synergistic across nearly all doses that were evaluated (Fig. 6a–d).

## Discussion

Despite the rarity of BCR pathway mutations in WM and their absence in many patients with ABC DLBCL, there are indications that chronic active BCR signaling is occurring in these entities. In previous studies, we identified that BTK, a downstream contributor to BCR signaling, was activated by mutated *MYD88* through HCK and was incorporated into the “Myddosome” signaling complex that supported NFκB pro-survival signaling^5,6^. These findings enabled the investigation and fast-track approval of ibrutinib, a pleiotropic kinase that targets both BTK and HCK for WM. However, the activation of BTK by mutated MYD88 did not provide an explanation for SYK activation, an upstream BCR member that is activated in *MYD88*-mutated WM patient cells^17^. Moreover, the finding that fostamatinib triggered apoptosis of *MYD88*-mutated WM cells, suggested an important function for SYK in MYD88 pro-survival signaling^20^. We therefore investigated a role for SYK as a mediator of TLR/BCR cross talk in *MYD88-*mutated lymphomas.

**Fig. 7:**
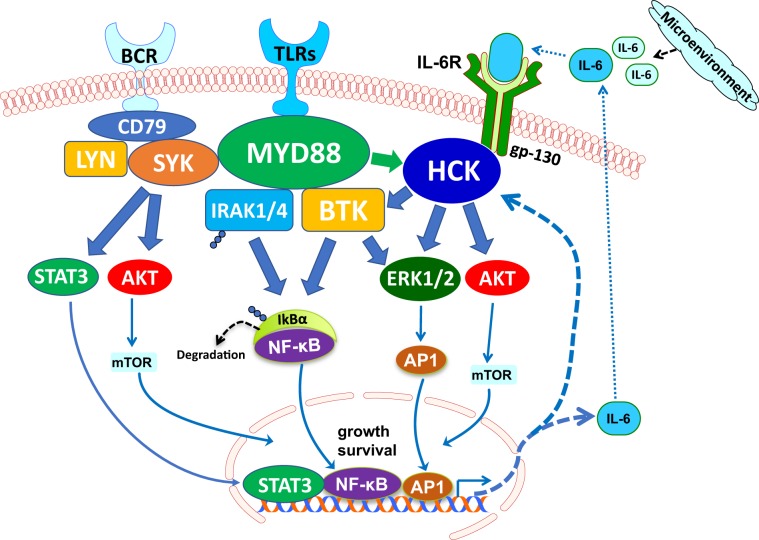
Pro-survival signaling mediated by mutated MYD88. Mutated MYD88 triggers assembly of the “Myddosome” that includes activated BTK and IRAK4/IRAK1 that transactivate NFκB. Mutated MYD88 also transcriptionally upregulates and transactivates through IL-6 the SRC family member HCK, that triggers activation of BTK itself, as well as AKT, and ERK. Mutated MYD88 also activates SYK, a component of the “Myddosome” that triggers STAT3 and AKT.

We observed that SYK in its active form was incorporated into the “Myddosome” signaling complex by both co-immunoprecipitation and co-localization experiments. Furthermore, by knockdown studies as well as use of SYK inhibitors, we validated the importance of SYK as an essential pro-survival molecule in *MYD88*-mutated lymphoma cells. The findings are consistent with those recently reported by Phelan et al.^26^, who described a MYD88-TLR9-BCR(IGM) (MY-T-BCR) super complex in MYD88-mutated tumor cells. Importantly, we found that SYK was activated by mutated MYD88, and served as an intermediary to trigger pro-survival STAT3 and AKT, but not BTK signaling. The latter finding is consistent with our earlier work that showed BTK activation was dependent on mutated *MYD88*^5^. Our current observations depict SYK and BTK, classically viewed as hierarchal components of BCR signaling, as “Myddosome” recruits that serve independent roles in propagating MYD88/TLR pathway signaling. Taken together, the findings add to the complexity and reach of mutated MYD88 to drive multiple pro-survival pathways that lead to NFκB, AKT, ERK, and STAT3 activation (Fig. 7).

The identification of SYK as a pro-survival signaling intermediary for mutated MYD88 may also explain the lack of complete responses reported with ibrutinib in *MYD88*-mutated B-cell malignancies, since SYK unlike BTK or HCK is not a target of these agents^6–11^. Importantly, the combination of SYK inhibitors with ibrutinib yielded synergistic lethality in *MYD88*-mutated lymphoma cells. These findings provide support for clinical trials combining SYK inhibitors with ibrutinib for *MYD88*-mutated lymphomas. Such an approach may be particularly suited to patients with both *MYD88* and *CD79* activating mutations since SYK remained active despite *MYD88* inhibition in *MYD88/CD79* double-mutated ABC cell lines. SYK inhibitors would therefore block upstream SYK activation mediated by activating CD79 mutations in these double-mutated tumors. Several SYK inhibitors, including fostamatinib, entospletinib, TAK-659, and HMPL-23 are currently in development for hematological and oncological indications. Fostamatinib was recently approved for the treatment of chronic ITP and showed significant single agent activity in previously treated CLL^27^. Entospletinib, a more selective SYK inhibitor, has also shown notable activity in previously treated CLL, with good tolerance and is currently being evaluated in combination with obinutuzumab in CLL, SLL, and NHL (NCT 03010358)^22,28^. The SYK inhibitor TAK-659 has also shown activity and good tolerance in FL, and DLBCL, including transformed cases^29^. A study evaluating TAK-695 in combination with chemotherapy is currently underway in high-risk DLBCL (NCT 03742258). Responses to the SYK inhibitor HMPL-3 have also been observed across multiple B-cell lymphomas, including WM^30^.

In summary, we have identified activated SYK as an integral component of the mutated *MYD88* signaling apparatus, and an intermediary for AKT and STAT3 pro-survival signaling. Combined use of ibrutinib and SYK inhibitors produces synergistic lethality in *MYD88*-mutated B cells, including those with accompanying *CD79* activating mutations. The findings provide a framework for the clinical investigation of ibrutinib with SYK inhibitors in *MYD88*-mutated lymphomas.
